# Supplementary material for: Restoring Osteochondral Defects through the Differentiation Potential of Cartilage Stem/Progenitor Cells Cultivated on Porous Scaffolds
Source: Cells. 2021 Dec 14;10(12):3536. doi: 10.3390/cells10123536 (PMC8700224; doi:10.3390/cells10123536)

Figure S1. A schematic diagram of the studied design

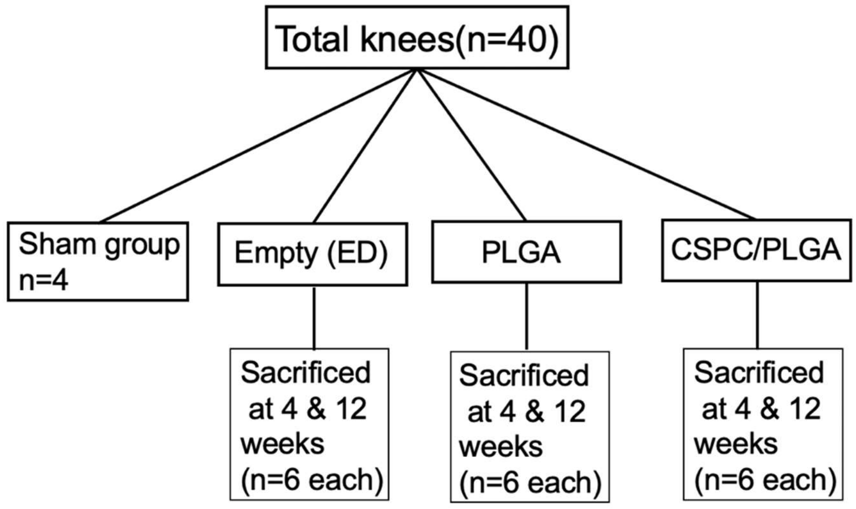

Table S1: A modified Wayne's grading scale scoring system for gross appearance

Macroscopic Appearance	Description	Points
1. Coverage	>75% fill	4
	50-75% fill	3
	25-50% fill	2
	<25% fill	1
	0% fill	0
2. Tissue Color	Normal/whitish	4
	25% yellow/brown/reddish/white	3
	50% yellow/brown/reddish/white	2
	75% yellow/brown/reddish/white	1
	100% yellow/brown/reddish/white	0
3. Surface (smooth level)	Normal	4
	Smooth but raised	3
	25-50% irregular	2
	50-75% irregular	1
	> 75% irregular	0
Total score		12

Figure S2: Proliferative potential of (A) chondrocytes or (B) osteoblasts cultured in extracellular vesicle (EV) derived medium from CSPCs or IFPs.

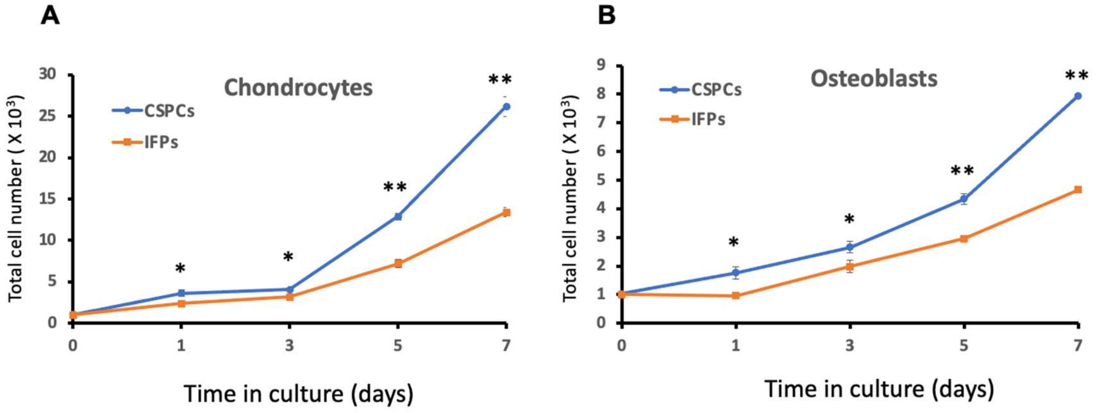

Supplement: Supplementary file 1 [file cells-10-03536-s001.zip › cells-1450421-supplementary.pdf]
